# Quality of anticoagulant control and patient experience associated with long-term warfarin in Canadian patients with non-valvular atrial fibrillation: A multicentre, prospective study

**DOI:** 10.1371/journal.pone.0284425

**Published:** 2023-04-18

**Authors:** Rita Selby, Lisa Kaus, Faith Sealey, Marika Koo, Sameer Parpia, Brian Chan, Soo Jin Seung, Carole Bradley, Rachel Strauss, Nicole Mittmann

**Affiliations:** 1 Department of Medicine, Sunnybrook Health Sciences Centre, University of Toronto, Toronto, Ontario, Canada; 2 Department of Laboratory Medicine and Pathobiology, University of Toronto, Toronto, Ontario, Canada; 3 Department of Clinical Pharmacology, Health Outcomes and Pharmacoeconomics (HOPE) Research Centre, Sunnybrook Health Sciences Centre, Toronto, Ontario, Canada; 4 Department of Oncology, Department of Health Research Methods, Evidence and Impact, McMaster University, Hamilton, Ontario, Canada; 5 KITE—Toronto Rehab Institute, University Health Network, University of Toronto, Toronto, Ontario, Canada; 6 Institute of Health Policy, Management and Evaluation, University of Toronto, Toronto, Canada; 7 Boehringer Ingelheim Canada, Burlington, Ontario, Canada; 8 ICES, Toronto, Ontario, Canada; 9 Sunnybrook Research Institute, Sunnybrook Health Sciences Centre, University of Toronto, Toronto, Ontario, Canada; 10 Department of Pharmacology and Toxicology and Institute for Health, Policy and Evaluation, University of Toronto, Toronto, Ontario, Canada; BSMMU: Bangabandhu Sheikh Mujib Medical University, BANGLADESH

## Abstract

**Background:**

Despite the fact that direct oral anticoagulants (DOACs) are favoured over warfarin for stroke prevention in patients with non-valvular atrial fibrillation (NVAF), physicians need to maintain competence in using and monitoring warfarin since many patients have contraindications or other barriers to using DOACs. Unlike DOACs, warfarin therapy requires regular blood testing to ensure that it is within a target range to ensure efficacy and safety. There is limited real-world data on the adequacy of warfarin control and the cost and burden of monitoring warfarin therapy in Canadian NVAF patients.

**Objectives:**

In a large cohort of Canadian patients with NVAF on warfarin we assessed time in therapeutic range (TTR), determinants of TTR, process of care, direct costs, health related quality of life and loss of work time and productivity related to warfarin therapy.

**Methods:**

Five hundred and fifty one patients with NVAF, either newly initiated or stable on warfarin were prospectively enrolled across 9 Canadian provinces from primary care practices and anticoagulant clinics. Participating physicians provided baseline demographic and medical information. Patients completed diaries for 48 weeks, capturing information about International Normalized Ratio (INR) test results, test locations, process of INR monitoring, direct costs of travel, health-related quality of life and work productivity measures. TTR was estimated using linear interpolation of INR results and linear regression used to investigate associations between TTR and factors (defined a priori).

**Results:**

Four hundred and eighty (87.1%) patients had complete follow-up with an overall TTR of 74.4% based on 7,175 physician-reported INR values from 501 patients. 88% of this cohort were monitored through routine medical care (RMC). The average number of INRs per patient during the 48-week period was 14.1 (standard deviation (SD) = 8.3) tests with a mean duration of 23.8 (SD = 11.1) days between tests. We did not find a relationship between TTR and age, sex, presence of major comorbidities, patient’s province of residence or rural vs. urban residence. 12% of patients monitored through anticoagulant clinics had significantly better TTR than patients monitored through RMC (82% vs. 74%; 95% confidence interval: -13.8, -1.2; p = 0.02). Health related quality of life utility values were high and remained consistent throughout the study. The majority of patients reported no impact on either work productivity or impairment of regular activities due to being on long-term warfarin treatment.

**Conclusions:**

We showed excellent overall TTR in an observed Canadian cohort, with monitoring through a dedicated anticoagulant clinic being associated with a statistically and clinically significant improvement in TTR. The burden of warfarin therapy on patients’ health related quality of life or daily work and activities was low.

## Introduction

Oral anticoagulant therapy (OAT) is widely used for the prevention and treatment of both venous and arterial thromboembolism (TE). Duration of OAT can range from 3 months to life-long depending on the indication, such as stroke prevention in atrial fibrillation or with prosthetic heart valves, venous thromboembolism (VTE) prophylaxis or treatment [1–3]. Non-valvular atrial fibrillation (NVAF), the most common indication for long-term OAT affects approximately 1 to 4% of individuals worldwide with prevalence increasing with age [4]. Ischemic strokes and systemic embolism occur in approximately 5–7% of NVAF patients per year, an incidence five times higher than in patients who do not have AF. (4[4,5] OAT reduces this risk by 60–65%, to 1–2% per year, while reducing mortality in these patients (even when compared with antiplatelet therapy) and has become the mainstay of stroke prevention in NVAF [5–7].

World-wide randomized controlled trials (RCTs) have compared various direct oral anticoagulants (DOACs) to Vitamin K antagonists such as warfarin and concluded that these agents are at least as efficacious as well controlled warfarin in this setting [1,8]. These have largely replaced warfarin for many indications requiring short and long term anticoagulation. However, warfarin is still required for stroke prevention in AF patients who are unable to take DOACs due to drug interactions, or have financial barriers such as lack of access to drug insurance programs.

The narrow therapeutic index and various drug and food interactions require patients on warfarin to undergo regular dose adjustments based on the International Normalized Ratio (INR). The therapeutic target for most indications, including stroke prevention in NVAF, is around 2.5 (range between INR of 2–3) [9]. An INR lower than the target therapeutic range can increase a patients’ risk of recurrent thromboembolic (TE) events and a high INR can increase the risk of bleeding [10–12]. In patients with AF, moderate to good control of warfarin (generally defined as greater than 60% of the time spent in the therapeutic INR range) has been proven not only to significantly reduce TE events and death but also lower overall and major bleeding rates [10–14]. The country-specific data on the adequacy of warfarin control based on time in therapeutic range (TTR) is not described in many RCTs and there is a significant paucity of data on TTR in Canadian patients on long term warfarin who are monitored in a real-world setting, outside of clinical trials. The effect of macro, meso and micro system effects on the patient experience have not been studied prospectively.

### Objective

We conducted a prospective, observational, study of community dwelling Canadian patients with NVAF on warfarin therapy, over a period of 48 weeks to assess TTR, determinants of TTR, process of care, direct out-of-pocket costs, health related quality of life and loss of work time and productivity related to warfarin therapy.

## Methods

### Patients

Adult patients with a diagnosis of NVAF, on stable warfarin therapy, were recruited from family and specialist practices, and anticoagulant clinics from April 2008 to July 2012 in 9 Canadian provinces: British Columbia, Saskatchewan, Manitoba, Ontario, Quebec, Nova Scotia, Prince Edward Island, New Brunswick, and Newfoundland, with the final participant completing follow up in April of 2014. Oral anticoagulant therapy was defined as oral warfarin treatment. The planned recruitment was a convenience sample size of 500 to 600 patients with a follow up duration of 48 weeks, each requiring the completion of weekly diaries capturing both INR results a number of variables related to being on long-term warfarin treatment. Inclusion criteria were: (1) Patients receiving warfarin for NVAF who had been on warfarin for at least 1 year. Patients could have a second indication for long-term warfarin (e.g., secondary VTE prophylaxis, cardiomyopathy); (2) Equal or greater than 18 years of age. Exclusion criteria were: (1) Patients on a vitamin K antagonist other than warfarin (e.g. nicoumalone, phenprocoumon) (2) Inability to provide informed consent (3) Inability to participate for the entire duration of the study (4) Patients who were being monitored using Point-of-Care (POC) INR devices either through self-testing or testing in a health care facility (5) Patients participating in clinical trials of anticoagulant therapy and (6) Inability to communicate: speak, read and understand English or French.

### Recruiment

This study was approved by institutional and community research ethics boards in each participating province. Patients were recruited in two ways: (1) a list of Canadian physicians was obtained from MDSelect (www.MDselect.com) with an online directory of almost 82,000 Canadian physicians. A simple random sample of primary care physicians (N = 200) was generated from this list by province. This provincial list of 200 primary care physicians was then cross-referenced with a list of warfarin prescribers obtained from the study sponsor to increase the probability that the primary care physician who was contacted would be eligible to participate; (2) anticoagulant clinics operating in academic medical centers were contacted to participate. The goal was to recruit 80% of eligible patients through primary care and 20% through specialized anticoagulant clinics to approximate patterns of OAT care worldwide where the majority of warfarin is monitored outside of specialized settings [15]. We also differentially sampled patients per participating province based on the population estimates of that province. Physicians who agreed to participate in our study were responsible for initial screening of their eligible patients for enrollment, obtaining patient consent, patient demographics and baseline medical information, and providing this, as well as patient contact information to three central study coordinators at Sunnybrook Health Sciences Centre (LK, FS, and MK). Patients provided written informed consent for participation in the study. Once enrolled, coordinators would then contact the patient, mail the weekly diaries to the patient, and provide phone-based instructions and support in filling out the diaries both initially, and then monthly over the course of the study.

### Study variables and data collection

The enrolled patients reported on a number of variables each week that included dates of INR tests, test locations, results of INR tests if known, who provided them with test results, how long they had to wait for test results and whether warfarin dose was adjusted on this basis. They also provided data on approximate distance to INR testing location, modes of transportation with associated out-of-pocket transportation and parking costs, health-related quality of life and lost productivity. Health-related quality of life was measured using the EQ-5D-3L questionnaire which is an established and well validated questionnaire used in many therapeutic settings [16]. It asks the patient to rate their functioning across five dimensions: mobility, self-care, usual activities, pain/discomfort and anxiety/depression. Responses to EQ-5D-3L questionnaire were converted to utility values, a measure of health preference bounded between 0 (death) to 1 (perfect health). The conversion tool was based on a Canadian EQ-5D valuation study [17]. Lost productivity was measured using the Work productivity and Activity Impairment (WPAI) Instrument. (http://www.reillyassociates.net/WPAI_General.html). This instrument measures the lost productivity (both in terms of work time missed or reduced productivity) that results from illness and activity impairment. Patients mailed completed diaries each month to the study co-ordinating site that were entered into a centralized database after direct resolution of queries with patients over the phone. Patients were compensated for the time taken to complete each week’s dairies at a nominal amount of $4 CAD. Participating physicians or clinics also provided all INR laboratory reports for the enrolled patient for the study duration to the coordinating site which were also entered into the database. All electronic data entries were checked by a second study coordinator for accuracy.

### Statistical analysis

Baseline characteristics, utility values, loss in work productivity, mode of transportation and transportation costs for INR testing of the patients were described with proportions and means for categorical and continuous variables, respectively. Patients with more than one available INR value were included in the analysis of TTR. TTR was estimated using the Rosendaal method which assumes that the unobserved INR levels between observation times follow a linear trajectory [18]. INR data were collected from laboratory reports provided by participating physicians. INRs between 1.96 and 3.04 (for a target range of 2.0 to 3.0) were considered therapeutic because some laboratories reported INRs rounded to 1 decimal (for example, 1.96 was reported as 2.0). Multivariable linear regression was used to investigate associations between TTR and factors that were identified a priori. Although patients also provided INR results as entries in their diaries, this data was not used to calculate TTR since there may have been missing values when results were not provided to the patient or inaccursacy when patients recorded them in their diaries (communication or transcription errors). Statistical analysis was performed using SAS 9.2 (Cary, NC). R statistical software was used for the statistical analysis of utility values, work productivity, mode and cost of transportation for INR testing. (R Core Team (2019) R: A language and environment for statistical computing. R Foundation for Statistical Computing, Vienna, Austria). Patient diaries were used to calculate costs related to transportation and parking. Utility scores (between 0 and 1) were determined by converting EQ-5D scores while work impairment was reported as a percentage.

## Results

Five hundred and fifty-one patients were enrolled between April of 2008 and July of 2012. Baseline characteristics of the population are described in Table 1. The mean age of the cohort was 76 years (Standard deviation (SD) = 9 years). Sixty percent were male and 40% female. Hypertension was the most prevalent co-morbidity (67%), followed by ischemic heart disease (30%), and diabetes (26%). The study participants were predominantly from Ontario (38%), followed by Manitoba (13%) and Nova Scotia (13%). Participation by province is shown in Table 1. Eighty-eight percent of patients had their warfarin managed through routine medical care (RMC) while 12% attended dedicated anticoagulant clinics (AC). Postal code analysis of participants’ residence indicated that 78% of patients were from urban areas and 22% from rural.

**Table 1:** Baseline characteristics.

Variable	All Patients (n = 551)
Age (years): *mean (SD)*	76 (9)
Sex: *n (%)*	
Male	333 (60)
Female	218 (40)
Co-morbid illnesses: *n (%)*	
Cerebrovascular disease	93 (17)
Other neurological disorder	27 (5)
Ischemic heart disease	164 (30)
Other heart disease	132 (24)
Diabetes	141 (26)
Hypertension	367 (67)
Venous thromboembolism	27 (5)
Cancer	96 (17)
Rheumatological disorder	87 (16)
Gastrointestinal disorder	79 (14)
Province: *n (%)*	
Ontario	207 (38)
Manitoba	73 (13)
Nova Scotia	70 (13)
British Columbia	57 (10)
New Brunswick	53 (10)
Quebec	40 (7)
Prince Edward Island	20 (4)
Saskatchewan	19 (3)
Newfoundland	12 (2)

Of the 551 patients enrolled, 480 participants (87.1%) completed the entire study. Of the 71 patients (12.9%) who did not, there were 45 drop-outs, 9 deaths and 17 who discontinued warfarin making them ineligible to continue.

The mean number of INR tests per patient during the 48-week period was 14.1 (SD = 8.3) tests with a mean duration of 23.8 (SD = 11.1) days between tests over 281.2 (SD = 77.1) follow up days. Eighty-two percent of patients received their INR test results by telephone communication. Mean wait time to get results was 1.4 (SD = 2.3) days after the test. Fifty-two percent of the INR tests were performed at non-hospital-based community laboratories. Of the 8,178 INR testing visits that were undertaken by participants during the study follow up period, patients driving themselves to their appointments was the most frequent mode of transportation reported (69%, n = 5,602) followed by being driven by family or a friend (19%, n = 1,591) (Table 2A). More than 90% of study participants reported on the out-of-pocket costs of transportation to their INR testing locations, and over 95% reported on costs of parking (Table 2B). Transportation costs were between $1 and $10 CAD for 46% of INR testing visits (n = 3,760) and $0 CAD for 36% of visits (n = 2,933). Most INR testing visits did not incur any direct parking costs to the participants (77%, n = 6,289 visits), while 18% of visits (n = 1,503) cost participants between $1 and $10 CAD. Less than 10% and 1.3% of reported visits incurred more than $10 CAD in transportation and parking respectively.

**Table 2:** a. Transportation modes used by study participants when travelling for INR testing visits (n = 8,178 visits). b. Direct costs of transportation and parking for INR testing visits during study follow-up period (n = 8,178 visits).

Mode of transportation	N (%)
Drove	5,602 (69)
Driven by family/friend	1,591 (19)
Walked	340 (4)
Other	287 (3.7)
Taxi	190 (2.3)
Public transportation	168 (2)

Cost categories	Transportation to INR visits N (%)	Cost of parking for INR visits N (%)
$0	2,933 (36)	6,289 (77)
$1-$10	3,760 (46)	1,503 (18)
$11-$20	481(5.9)	89 (1)
More than $20	219 (2.7)	25 (0.3)
Did not report	785 (9.6)	272 (3.4)

Five hundred and seven patients had at least one INR reported by their physician (total of 7,181 INR tests reported). Six patients with only a single INR test reported were excluded from this analysis. The overall TTR was 74.4% (SD = 20.5) based on 7,175 physician-reported INR values from 501 patients. The distribution of TTR by various strata is shown in detail in Table 3A and 3B. Most patients (73%) had TTR over 65%, with 47% achieving a TTR of over 80%. A very small proportion of this cohort (only 7%) had poor TTR defined as less than 40% and only 15% had TTR in the range of 40–59%. Analysis of predictors of TTR is summarized in Tables 4 and 5. We did not observe evidence of a relationship between TTR and age, sex, presence of major co-morbidities such as cerebrovascular disease, cardiovascular disease or cancer. (Tables 4 and 5) Similarly, the effect of patient’s province of residence or rural vs. urban residence was also inconclusive. (Tables 4 and 5) The only predictor of improved TTR was if patients were monitored through an anticoagulant clinic compared to RMC (82% vs. 74%; p = 0.02). (Table 5) This however included only 12% of the study patients.

**Table 3:** a. Distribution of TTR based on poor, sub-optimal, good and excellent control. b. Distribution of TTR based on poor, fair and good control.

Time in Therapeutic Range (TTR) (%)	All patients, (n = 501) n (%)
< 40	33 (7)
40–59.9	73 (15)
60–79.9	157 (31)
>80	238 (47)
Time in Therapeutic Range (TTR) (%)	All patients, (n = 501) *n (%)*
< 40	33 (7)
40–65	103 (20)
>65	365 (73)

**Table 4:** Summary of Time in Therapeutic Range by factors (TTR) (n = 501).

Characteristic	n	TTR *Mean (SD)*
Sex:		
Male	299	75 (20)
Female	202	74 (21)
Cerebrovascular disease:		
Yes	84	74 (21)
No	416	75 (20)
Ischemic heart disease:		
Yes	144	72 (20)
No	355	75 (21)
Other heart disease:		
Yes	115	74 (19)
No	385	75 (21)
Cancer:		
Yes	86	77 (18)
No	414	74 (21)
Residential Location:		
Urban	389	75 (20)
Rural	112	73 (22)
Setting:		
Routine medical care	444	74 (21)
Anticoagulant clinic	57	82 (14)
Province:		
British Columbia	53	75 (16)
Manitoba	65	69 (21)
New Brunswick	50	76 (18)
Newfoundland	11	83 (19)
Nova Scotia	65	70 (22)
Ontario	180	74 (22)
Prince Edward Island	19	87 (12)
Quebec	39	83 (15)
Saskatchewan	19	70 (29)

**Table 5:** Results from multivariable linear regression (Outcome = TTR, n = 501).

Variable	Estimate (95% Confidence Interval)	p-value
Age	0.0 (-0.2, 0.2)	0.73
Sex: *Male vs*. *Female*	0.2 (-3.6, 4.0)	0.91
Cerebrovascular disease: *Yes vs*. *No*	-0.2 (-5.1, 4.7)	0.94
Ischemic heart disease: *Yes vs*. *No*	-3.3 (-7.3, 0.8)	0.11
Other heart disease: *Yes vs*. *No*	-0.6 (-5.0, 3.7)	0.77
Cancer: *Yes vs*. *No*	3.0 (-1.9, 7.9)	0.22
Residence: *Urban vs*. *Rural*	1.6 (-2.9, 6.1)	0.50
**Setting:** *RMC vs. *AC*	**-7.5 (-13.8, -1.2)**	**0.02**
Province: Ref = Ontario & Quebec*		
British Columbia	-0.1 (-6.6, 6.5)	
Nova Scotia, Prince Edward Island, New Brunswick, Newfoundland	1.3 (-3.3, 6.0)	
Saskatchewan, Manitoba	-4.9 (10.3, 0.6)	

*RMC–Routine medical care, AC–Anticoagulant clinic.

Health-related quality of life utility values were collected monthly and remained consistent throughout the study follow-up period ranging from a mean of 0.82 (SD = 0.16) to 0.84 (SD = 0.16) (median 0.82–0.83)–Table 6. At study baseline follow-up, of the participants that completed the WPAI instrument, a total of 15% of the study participants (n = 86) were currently employed during the study follow-up period. Of the participants employed, the mean percentage of work time lost and impairment of productivity at work due to illness were 1.6% (SD = 7.1%) and 8.3% (SD = 25.4%) respectively, (median 0% for each measure). In total, the overall work impairment from both work time lost or impairment of productivity while working due to illness was a mean 4.8% (SD = 14.0%) and median of 0%. The entire study cohort reported a mean and median of 8.3% (SD = 25.4%) and 0% respectively of impairment of regular daily activities due to illness.

**Table 6:** Mean and median utility values stratified by study follow up period.

Follow-up period (month)	n	Mean (standard deviation)	Median (interquartile range)
1	516	0.834 (0.166)	0.835 (0.274)
2	296	0.830 (0.158)	0.826 (0.274)
3	458	0.838 (0.157)	0.843 (0.274)
4	441	0.840 (0.147)	0.836 (0.274)
5	416	0.826 (0.164)	0.836 (0.274)
6	404	0.827 (0.155)	0.836 (0.274)
7	392	0.824 (0.157)	0.836 (0.274)
8	389	0.825 (0.156)	0.836 (0.274)
9	384	0.825 (0.158)	0.817 (0.274)
10	380	0.822 (0.160)	0.817 (0.274)
11	367	0.822 (0.162)	0.826 (0.274)
12	357	0.824 (0.154)	0.817 (0.274)

## Discussion

We report findings from a pan-Canadian, real-world, prospective, longitudinal study that has quantified time in therapeutic range, determinants of TTR, direct costs to patients and impact on quality of life and work productivity in a large cohort of NVAF patients on warfarin. We found an overall TTR of 74.4% with no statistically significant associations between TTR and potential predictors, other than the setting in which the patient is monitored. As well, patients reported no change in their quality of life over the 48 week study period and no significant impact from their warfarin treatment on either their work productivity (in the 15% of participants who were employed) or impairment of regular daily activities for the entire cohort.

In the last decade several DOACs have been compared to warfarin for stroke prevention after NVAF in worldwide randomized controlled trials [19–22]. Mean TTR for the warfarin comparison arms in these clinical trials has ranged from 55% to 83% and these differences have been country and health care system specific [19–25]. Where data are available specifically for Canadian patients enrolled in RCTs, their TTR has met the definition of good control with a mean of over 65% [23–26]. However, since these data are from patients managed in clinical trials, they do not reflect real world care of patients on warfarin.

Time in therapeutic range is a good surrogate marker for both efficacy and safety of warfarin [8,10–14,27,28]. Despite the fact that DOACs have become first line therapy for the prevention and management of thrombosis for many clinical indications, there is still an important role for warfarin when DOACs are contraindicated, for example in patients with valvular atrial fibrillation, severe renal insufficiency, mechanical heart valves, high-risk antiphospholipid antibody syndrome and concomitant medications that have a significant interactions with DOACs [29,30]. In addition, patients who cannot afford DOACs for long term therapy have no option other than warfarin which is significantly cheaper. Data such as ours, demonstrating good warfarin control being achieved outside of a clinical trial setting within our own health care environment is important in determining the effectiveness of warfarin control for patients that cannot take a DOAC for all the above stated reasons. Almost 88% of our study cohort contributed 46 weeks or more of study diaries resulting in almost an entire year of follow up with a very low rate of loss to follow up (71 patients;12.9%) due to warfarin discontinuation, death or dropping out from the study. Therefore an overall TTR of 74%, with most patients (73%) achieving a TTR of over 65%, and almost half of the cohort (47%) achieving a TTR of over 80%, can be considered good to excellent warfarin control, even better than what was achieved in clinical trial settings [19–22]. A very small proportion of this cohort (only 7%) had poor TTR defined as less than 40% and only 15% had TTR in the range of 40–59%. Comparable results were achieved in a small Canadian study by Wilson et al in 221 patients who required at least 3 months of warfarin and were randomized to warfarin management through RMC or hospital based anticoagulant clinics [31]. The overall TTR in this study in the RMC group was 76%. However, this was a randomized controlled trial and not a real-world setting like our study.

Limited, real world Canadian data have demonstrated variable but generally sub-optimal TTR on warfarin. A retrospective review conducted in 2007, compared oral anticoagulant care across 5 countries and suggested that RMC in Canada is suboptimal with poor documentation of care and lower TTR than countries that deliver anticoagulant care mostly through dedicated anticoagulant clinics [32]. Two small Canadian studies–a review of three primary care centres in Ontario with dedicated anticoagulant monitoring systems, and a cross sectional survey of physicians in Nova Scotia reported adequate knowledge on the part of physicians monitoring oral anticoagulant therapy, and adequate time in range respectively [33,34] A recent cross sectional study of 150 patients on warfarin from a family health network of 9 family physicians in southern Ontario who used a nurse-administered protocol for warfarin monitoring achieved an overall TTR of 58.76%—at the lower end of the target threshold [35]. A recent retrospective cohort study from the Canadian province of Alberta in over 40,000 patients with NVAF who were dispensed warfarin for over a month, and had 3 or more INR measurements found that only 41% of patients had good control with a mean TTR of over 65% [36]. Nearly a third of the original cohort of 57,669 patients had insufficient monitoring with less than 3 INR measurements in the first 6 months of their warfarin prescription which may have accounted for the worse TTR. In contrast, patients in our study had a mean of 14 INR measurements over approximately a one year study follow up period (on average monthly INR measurements) which could have explained the better warfarin control seen in our study. These few studies, however, contribute the only data evaluating TTR in Canadian patients on warfarin therapy.

Our study describes the determinants of TTR in Canadian patients. Although one can hypothesize that older patients with more comorbidities and / or mobility issues may have lower TTR, we did not find evidence of this relationship in our study. Neither did patients living in rural areas who may have restricted access to INR monitoring and timely care. There was also no significant difference in mean TTR across the nine Canadian provinces that participated in this study. Age, sex, comorbidities, place of residence and province of residence as determinants of TTR in Canadian patients, have not been previously examined in a prospective, real world study.

When Canadian patients are managed through anticoagulant clinics compared to RMC, TTR is significantly improved, as was seen both in our study (AC vs RMC; 82% vs 74%) and the Canadian RCT by Wilson et al (AC vs RMC; 82% vs 76%) [31]. The effect of the setting in which warfarin care is delivered in improving TTR has been previously reported, with dedicated anticoagulant clinics being associated with higher TTR than routine medical care [15,37,38]. Anticoagulant clinic care was the only significant predictor of a higher TTR in our study although only 12% of our study cohort received care this way.

Only 15% of study participants were employed, and did not find that warfarin treatment was burdensome in terms of work time lost or impaired work productivity. As well, the entire cohort did not report significant impairment of regular daily activities due to warfarin treatment. Overall, the entire cohort reported a good health related quality of life that stayed consistent over the course of the study. When compared to recent age-specific utility norms of Canadians, the mean range of 0.82 to 0.84 in our study was even higher than 0.792 for the age range of 75–79 years [39].

A small number of studies have looked at health related quality of life in NVAF patients on long term anticoagulation. Older studies, both randomized trials [40,41], and observational studies [42–44], have not found a negative impact of long-term warfarin therapy on either patient-perceived heath status or quality of life in NVAF patients. More recently, a sub-analysis of the RE-LY RCT (a phase III study that compared two doses of dabigatran to warfarin) reported on health related quality of life in study participants using the EQ-5D questionnaire [45]. In this study, 1435 patients (982 on dabigatran and 453 on warfarin) recorded baseline utilities in the upper quartile of possible values, ranging from 0.805 [SD = 0.205] to 0.811 [SD 0.193], which remained stable over the course of one year of follow up in patients who did not have outcomes (strokes or major bleeds). Although this study did not specifically report on the Canadian participants in the trial, the utilities and their stability over a year for the entire cohort are similar to what we found in our real-life observational study. To our knowledge, these utilities as an estimate of quality of life have not been previously reported in community dwelling, Canadian NVAF patients on long-term warfarin.

Our study had several strengths. This is the first prospectively conducted study representing care across the majority of Canadian provinces over a long term period of follow up, and diverse care settings, to determine both TTR, determinants of TTR, process of care, direct costs and burden of warfarin therapy in NVAF patients. There were also several limitations. Firstly, since patients were invited to participate through their primary care physician or anticoagulant clinics and our TTR was similar to or better than that achieved in clinical trial settings, it is possible that there was a patient selection bias. More highly motivated patients with greater investment in their own care, more educated about their disease and treatments, proactive about keeping appointments, and with better overall function, may have been more likely to participate, potentially biasing the TTR results positively. In the same fashion, it is possible that physicians who were more invested in monitoring patients appropriately, were more likely to participate. Since most real world Canadian data is retrospective, and shows worse TTR than we reported it is possible that the lack of selection bias may suggest a more accurate reporting of the true TTR in Canadian patients. However retrospective data lacks many patient-level descriptors that our study was able to capture. Secondly, we were unable to assess associations between TTR with clinical outcomes. Although patients were asked to document visits to physicians, and hospitalizations or interventions for bleeds or thrombotic episodes in their weekly diaries, we were unable to objectively verify and adjudicate these clinical events due to inability to procure reliable source documentation from their primary care physicians. However, it is well accepted that TTR is a good surrogate measure for clinical outcomes, both bleeding and thrombosis [8,10–14,27,28]. Finally, we were unable to assess the effects of concomitant medications or diet and lifestyle choices on TTR, because of the inherent difficulties in recording the accurate start and stop times of medications or diets interacting with warfarin by patients, as well as the ability to accurately verify and analyze these data. In conclusion, in a large cohort of Canadian, community dwelling patients on warfarin for stroke prevention due to NVAF, we found good to excellent TTR, better than TTR in Canadian patients enrolled in RCTs. Most patients had their INR monitored through routine medical care and at least half, visited community based laboratories for INR tests. The only predictor of a significantly higher TTR in our study cohort was receiving care from a specialized anticoagulant clinic. Age, sex, presence of comorbidities or place of residence within Canada did not predict TTR. Although we could not adjudicate clinical outcomes, high TTR is an accepted surrogate outcome for both reduced thromboembolic events and bleeding in patients on long term warfarin therapy. These patients also reported a good quality of life and no significant impairment in either their work productivity or regular daily activities due to being on long-term warfarin despite its inherent laboratory monitoring requirements. This documentation of the process of delivering warfarin care, the direct costs to patients of monitoring warfarin, and their quality of life on long-term warfarin, should help guide both the conduct of future studies in this area, as well as inform national policies around the public funding of current and future oral anticoagulant medications that will be compared to warfarin.
